# Review on fat replacement using protein-based microparticulated powders or microgels: A textural perspective

**DOI:** 10.1016/j.tifs.2020.10.032

**Published:** 2020-12

**Authors:** Ben Kew, Melvin Holmes, Markus Stieger, Anwesha Sarkar

**Affiliations:** a Food Colloids and Bioprocessing Group, School of Food Science and Nutrition, Faculty of Environment, University of Leeds, Leeds, LS2 9JT, UK; b Division of Human Nutrition and Health, Wageningen University, PO Box 17, 6700 AA Wageningen, the Netherlands

**Keywords:** Fat replacer, Fat mimetic, Microparticulated, Plant protein, Tribology, Microgels

## Abstract

**Background:**

Due to the growing rise in obesity and food-linked diseases, the replacement of calorie-dense fat has been a key focus of food industries in the last few decades with proteins being identified as promising fat replacers (FRs).

**Scope and approach:**

This review aims to provide an overview of animal and plant protein-based FR studies that have been performed in the last 5 years. Protein isolates/concentrates, their microparticulated forms and protein microgels in model and real foods have been examined. Special emphasis has been given on the characterisation techniques that have been used to compare the full fat (FF) and low fat (LF) versions of the foods using FRs.

**Key findings and conclusions:**

Microparticulated whey protein (MWP) has been the preferred choice FR with some success in replacing fat in model foods and dairy applications. Plant proteins on the other hand have attracted limited research attention as FRs, but show success similar to that of animal proteins. Key characterisation techniques used to compare full fat with low fat products containing FRs have been apparent viscosity, texture profile analysis, microscopy, particle size and sensory properties with oral tribology being a relatively recent undertaking. Coupling tribology with adsorption techniques (muco-adhesion) can be effective to bridge the instrumental-sensory property gap and might accelerate the development cycle of designing low/no fat products. From a formulation viewpoint, sub-micron sized microgels that show shear-thinning behaviour and have boundary lubrication properties offer promises with respect to exploiting their fat replacement potential in the future.

## Highlights

• Milk protein has shown potential to act as fat replacer (FR) in model and real food.
• Microparticulated whey protein (MWP) is most commonly applied as FR in dairy foods.
• More recently plant proteins have gained increased research interests to act as FRs.
• Rheology, particle size and sensory techniques are used to assess properties of FRs.
• Microgels with promising lubrication properties can be a potential FR in the future.

## Graphical abstract

**Figure:**
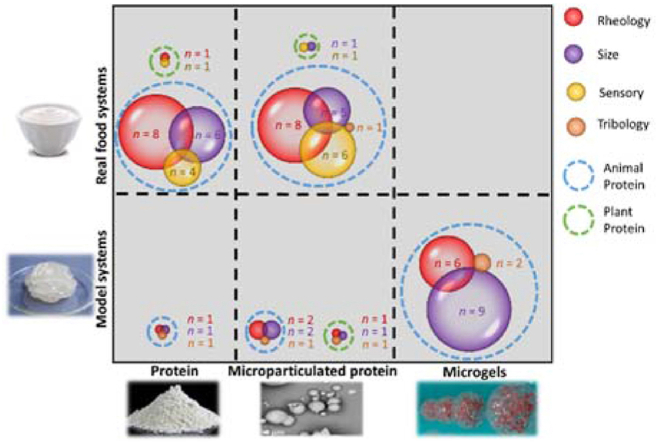

## 1 Introduction

Obesity is a growing health concern and socio-economic crisis globally (WHO, 2016). In the US, most adults are predicted to be overweight and 50% obese by 2030 consistently rising since 1999 (Wang et al., 2020). Obesity is related to a number of serious health issues such as cardiovascular diseases, cancer and type 2 diabetes with the latter accounting for nearly 9% of the overall National Health Service (NHS) budget in UK per year (Barron, Clark, Hewings, Smith, & Valabhji, 2018). Excessive consumption of calories particularly in the form of high fat western diets drive weight gain, with fat being the densest source of calories (9 kcal per gram) having more than double that of the carbohydrates and proteins. As a consequence, policy-makers and consumers are addressing dietary and associated health issues and, consequently food industries are attempting to accelerate their innovations in creating lower calorie food products (Bigliardi & Galati, 2013).

Reducing fat in foods is one of the potential strategies to decrease calorie intake significantly and consequently, LF versions of foods are gradually populating the supermarket shelves. However, many if not most, of these products are inferior in taste, texture and appearance as compared to their FF counterparts and thus do not thrive in the marketplace. Hence, there has been motivation in the food industry and research community to design fat replacers that mimic the functional and sensory properties of fat. These include two classes of materials: fat substitute (FS) and fat mimetic (FM) ingredients, with an attempt to replicate the physicochemical and sensorial properties of fat in food products. Typically, FS involves direct replacement of fat with a substance that attempts to provide similar organoleptic properties to fat, these can be synthetic in nature or structured lipid moeties that provide little to no calories (O'Connor & O'Brien, 2016). A classic example is ‘Olestra’, patented by Procter and Gamble Co (Jandacek & Letton, 1987). which is formed by esterification of sugars and long chain fatty acids which provides a Non-Newtonian pseudoplastic flow behaviour comparable to fats. Olestra behaves similar to those of conventional triglycerides but is resistant to lipolysis and, thus, serves as a non-calorific replacement for fats for designing LF foods. Other commercially formulated FS also include ‘Salatrim’ (Smith, Finley, & Leveille, 1994) and ‘Caprenin’ (William, Josef, & Joke, 1993), latter being designed as a cocoa butter replacement for use in confectionary applications. These have also been utilised in LF baked and dairy products. However, the use of FS has been limited owing to possible associations with abdominal cramping and other side effects (Hunt, Zorich, & Thomson, 1998), which has led to Caprenin being withdrawn and Olestra being prohibited for sale in the EU and other markets. FMs are substances that are used to mimic the specific microstructural, physicochemical and/or sensory properties of fat by using biocompatible and biodegradable carbohydrates and/or proteins either in their native form, in aggregated state or in the form of biopolymeric particles, physical complexes, either individually or in combination (Lucca & Tepper, 1994). In general, the FMs studied in literature can function by four potential mechanisms. These include 1) thickening the food matrix to replicate the rheological properties of fat, 2) mimicking the microstructure of emulsified fat droplets, 3) match the fat droplet-matrix interaction using suitable processing aids and capitalising colloidal interactions (pH, temperature, ions *etc.*), and 4) replicating the oral tribological properties of fat *i.e.* fat-oral surface interactions.

**Fig. 1:**
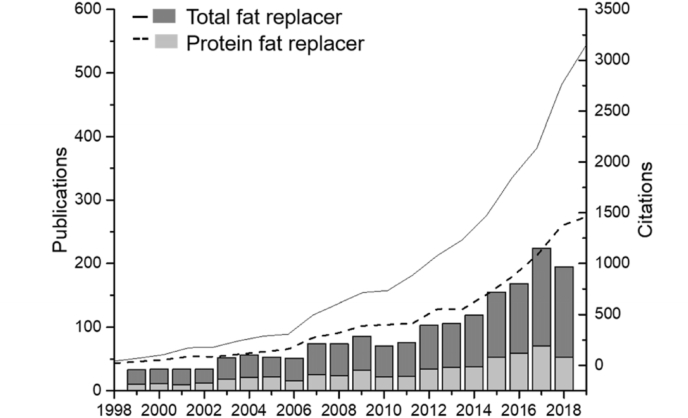
Number of publications (bars) and citations (lines) of fat replacers (black bar, solid line) and protein-based fat replacers (white bar, dashed line) using search engine, Web of science (ISI) from 1999 to 2018.

Proteins are often considered as a suitable macronutrient to replace fat as they contribute to only 4 kcal per gram (Benelam, 2009; Veldhorst et al., 2009) with more satiation, per calorie, compared to other macronutrients (Benelam, 2009; Gerstein et al., 2004; Hoek, 2010; Westerterp-Plantenga, 2008). In fact, the protein content of foods correlates positively with Satiety Index scores (Holt et al., 1995). This benefit has been used by scientific community for considering protein based FRs. In addition, protein is also a highly tuneable structuring agent by virtue of its responsiveness to pH, ions, temperature and enzymes. Fig. 1 shows the reported bibliographic data for FRs and protein-based FRs showing the growth of scientific literature and citations with the highest number of publications being produced since 2017, highlighting the topical nature and importance of this field in the period (1998–2018). A substantial yearly increase can be observed from 2012 for both total FR and protein-based FRs, with the latter contributing to nearly one-third of the total FR publications to-date indicating this as a priority area in the food science community.

This review aims to assess the progress in the last 5 years in the design and application of protein-based fat replacement strategies in model and real food systems. We briefly discuss the protein concentrates of animal and plant origin followed by a strong emphasis on microparticulated forms of proteins that have been used as FRs. We have focussed on specific characteristics (*e.g.* size, viscosity (*η*), firmness, water holding capacity (WHC), colour, coefficient of friction (*μ*), sensory properties) that can be useful to compare FF and FR-containing LF versions of the product. We have subsequently examined applications of these FRs in some food products and discuss the degree of fat replacement that can be achieved without compromising sensorial properties. We highlight several characterisation techniques that can be employed to bridge the gap between instrumental and sensory techniques to accelerate the development cycle of FR and possibly create FRs of just-right mouthfeel properties to mimic fat in real food products. Finally, we provide future perspectives on protein-based microgels that have been substantially characterised in literature in terms of physicochemical properties and which possess the potential to mimic fat but require further research attention with respect to fat replacement. Outside the scope of the present review are carbohydrate-related FRs that do not include protein as a component (Peng & Yao, 2017) as well as structured lipids as FSs (Marangoni, van Duynhoven, Acevedo, Nicholson, & Patel, 2020). Also protein-polysaccharide based structures and their uses as fat replacers are outside the scope of this review (Goh, Sarkar, & Singh, 2008; Weiss, Salminen, Moll, & Schmitt, 2019). Insights into product-specific replacement of fat can be found in some recent elegant reviews, such as use of microparticulated whey protein in dairy texture improvement (Ipsen, 2017), or employment of FR in ice cream (Akbari, Eskandari, & Davoudi, 2019), oil-based condiments (Ma & Boye, 2013), meat products (Varga-Visi & Toxanbayeva, 2016) and baked foods (Colla, Costanzo, & Gamlath, 2018). Table 1 lists all the abbreviations used in this review.

**Table 1:** Abbreviations and symbols.

Acronyms/Symbols	Full form
BSA	Bovine serum albumin
CNF	Cellulose nanofibre
EWP	Egg white protein
FF	Full fat
FM	Fat mimetic
FR	Fat replacer
FS	Fat substitute
HPC	Hemp protein concentrate
HTST	High temperature short time
LF	Low fat
MCP	Microparticulated canola protein
MEWP	Microparticulated egg white protein
MHP	Microparticulated hemp protein
MSP	Microparticulated soy protein
MWP	Microparticulated whey protein
NWP	Nanoparticulated whey
N/D	Native/denatured
NF	No fat
OA	Overall acceptability
PWP	Polymerised whey protein
PPI	Pea protein isolate
QCM-D	Quartz crystal microbalance with dissipation
SPI	Soy protein isolate
SEM	Scanning electron microscopy
TPA	Texture profile analysis
WHC	Water holding capacity
WPI	Whey protein isolate
WPM	Whey protein microgels
*α*-la	*α*-lactalbumin
*β*-lg	*β*-lactoglobulin
G′	Storage modulus
G″	Loss modulus
tan(*δ*)	Loss factor
*η*	Viscosity
*K*	consistency coefficient
*n*	Flow behaviour
*μ*	Coefficient of friction

## 2 Protein isolates/concentrates as fat replacers

Protein-based powders fall into two main categories, those containing below 90 wt% protein are known as concentrates, whereas, above are protein isolates (Nehete, Bhambar, Narkhede, & Gawali, 2013). Protein concentrates and isolates possess a number of functional properties including gel forming, emulsifying, foaming, WHC *etc.* Among these, the WHC and viscosity of proteins are considered as key parameters for FR that affects colour, texture and other sensory properties, latter are relevant for FR purposes (Zhang, McCarthy, Wang, Liu, & Guo, 2015).

Most recent FR studies using protein as concentrates or isolates have been performed on dairy products such as milk (Olivares, Shahrivar, & de Vicente, 2019; Protte, Weiss, Hinrichs, & Knaapila, 2019), yoghurt (Fang, Shen, Hou, & Guo, 2019), ice-cream (Liu, Wang, Liu, Wu, & Zhang, 2018b) and cheese (El-Aidie, I Ghita, M El-Dieb, & Elgarhi, 2019; Urgu, Türk, Ünlütürk, Kaymak-Ertekin, & Koca, 2019). As one might expect, whey protein has been seen to currently dominate the field of protein-based FRs (Protte et al., 2019). This is because whey protein, which is readily available as a by-product of cheese manufacturing process, has similar flavour profile and thus highly compatible with dairy applications (Lesme, Rannou, Famelart, Bouhallab, & Prost, 2020). More importantly, whey protein has been extensively characterised in literature in terms of its structural and functional properties allowing for fat replacement to be approached more mechanistically. For instance, in stirred goat milk yoghurt, removal of fat led to increased syneresis, a weaker body and a 50% reduction in the apparent viscosity (Costa, Frasao, Rodrigues, Silva, & Conte-Junior, 2016). By increasing the levels of whey protein in the formulation up to 6.8 wt% total protein in the LF yoghurts, the syneresis was significantly reduced being comparable to those of the FF version with viscosity increased in the LF version by four times, latter containing no added whey protein (Costa et al., 2016). Whey has also been incorporated into low fat cheese which can mimic the breaking up of the casein network, often achieved with fat (Danesh, Goudarzi, & Jooyandeh, 2018). This is hypothesised due to the WHC and formation of microscopic free pools of water which reduce hardness and firmness (3–4% WPI g/L milk) although at concentrations of 4–6% g/L milk hardness returns as high levels of whey compacts the protein matrix.

Processing of proteins has been often used as a powerful tool to improve the functionalities of proteins for fat replacement purposes. Whey protein is comprised of two main proteins, *β*-lactoglobulin (*β*-lg) and *α*-lacalbumin (*α*-la) typically at 50% and 20% (Alves & Tavares, 2019). Native *β*-lg contains a single thiol group capable of binding water. When denatured, at around 75 °C (Croguennec, Tavares, & Bouhallab, 2017) more reactive thiol groups are exposed (Torres, Murray, & Sarkar, 2017), which not only binds more water but also binds to adjacent protein thiol groups forming a continuous porous network and allow entrapment of water in food matrices resulting in higher WHC. By heating whey protein isolate (WPI) (85 °C for 30 min) at pH 8.5, lesser quantity of the protein (1.0 wt%) (Zhang et al., 2015) was needed to obtain similar viscosity (695 mPa.s) in LF goat milk yogurts as compared to those containing higher concentration (6.8 wt% total protein) of native whey protein (672 mPa.s) at the same shear rates (Costa et al., 2016).

Similarly, on addition of heat polymerised whey protein (PWP), fabricated using heat treatment of whey protein at varying temperature (70–90 °C/5–15 min) and pH conditions (pH 7.0–9.0) to no fat (NF) yoghurt, there was an increase in viscosity in NF yoghurt, similar to that of FF yoghurts (Fang et al., 2019). In other words, the viscosity of NF yoghurt containing PWP was significantly higher than those of NF yoghurts or yoghurts containing 1% fat. PWP also increased the firmness and improved WHC significantly as compared to NF yoghurt. The key mechanism proposed using confocal laser scanning microscopy imaging was that PWP acted as a ‘water-immobiliser’ resulting in formation of yoghurt gel with smaller pore size, which was not achieved in NF yoghurt, latter having a loose network with large sized pores. Additionally, PWP was assessed as having an astringent aftertaste (Fang et al., 2019), highlighting one of the key sensorial issues associated with protein as a FR. Besides heat treatment, the ratio of whey protein to casein can also play an integral role on rheological properties of the LF products. For instance, in high protein (8 wt%) set yoghurt, increasing the ratios of heated whey protein (75 °C/5 min): casein ratio to 25:75 (w/w) and 35:65 (w/w) in comparison to the control (whey protein: casein ratio = 10:90 w/w), respectively, resulted in less coarse, smoother, viscous, shinier product with desirable thickness (Jørgensen et al., 2015).

Besides conventional viscosity measurements, tribology measurements (measuring coefficients of friction) have also shown promising capability to distinguish iso-viscous commercial samples of FF and NF/LF yoghurts and cheeses, which were also discriminated based on their sensory properties. It was suggested that in these dairy products, FF products formed a continuous film of coalesced oil, which reduced the friction between polymeric surfaces in the tribological set up, this friction reduction was not achieved by the LF products (Laguna, Farrell, Bryant, Morina, & Sarkar, 2017). In this light, WPI has recently demonstrated promising lubrication properties using tribology testing in model thermally treated protein-based solutions (Zhu, Bhandari, & Prakash, 2019). Increasing the ratio of whey protein to casein (0/100–100/0 w/w) in 3.4% w/v heat-treated milk protein solutions were found to increase viscosity by 32% and enhance lubrication with μ being around 10% lower at sliding speeds >10 mm/s. The lower μ of the solutions with replacement of casein by WPI was attributed to the smaller particle size (46–168 nm) and spherical shape of globular WPI that were postulated to act as nanometric ‘ball bearings’ mimicking that of fat droplet-associated lubrication. In addition, the denaturation of WPI at high temperature (95 °C for 10min) and possible formation of disulfide linkages with *κ*-casein at the surface of casein micelles was also cited as a possible reason for the decreasing of the μ by formation of a lubricant layer that prevented adhesive contacts between the tribological surfaces. However, gradual increase in whey protein resulted in transparency and loss in opaqueness, a key appearance trait of high fat dairy foods (Zhu et al., 2019).

With the increasing environmental sustainability, health, animal-welfare and allergy concerns as well as the rising popularity of vegan diets, there has been burgeoning interests to shift towards use of alternative plant proteins in formulated food products. However, as far as FR is concerned, the impact of replacement of fat directly with plant protein concentrates/isolates alone is very limited. For example, soy protein isolate (SPI) has often been used to replace fat in sausages in the past, however, has been shown to be associated with off-flavour, texture alterations and reduction in overall acceptability (Nasonova & Tunieva, 2019). Nevertheless, successful studies have been conducted in combination of soy protein hydrolysate with xanthan gum (Liu, Wang, Liu, Wu, & Zhang, 2018b) to reduce ice cream fat by 50% and SPI with cellulose nanofibers (CNF) (Guo et al., 2018; Sun, Chen, Liu, Li, & Yu, 2015), where texture profile analysis (TPA) indicated no significant difference between SPI-CNF and control FF ice cream (Sun et al., 2015). Furthermore studies on understanding properties of dairy protein replacement by plant protein systems such as pea protein isolate (PPI), SPI and hemp protein concentrate (HPC) added at 1.4–2.8% have shown success to enhance the WHC by 1–8% leading to increased viscosities from 470 mPa s to 560 mPa s (Dabija, Codina, Anca, Sanduleac, & Lacramioara, 2018), whether such properties can be used to replace fat remains elusive in literature to date.

In summary, most systems of fat replacement using protein concentrates/isolates have been conducted using a dairy medium as applications with the replacement of dairy fat as the sole purpose. Therefore, high fat plant-based products, such as hummus, vegetable patties, vegan cheese which contain similar aromatic and taste profiles to plant proteins may be more compatible for testing replacement of fat with plant protein isolates/concentrates. Plant protein isolate/concentrates that have fat mimicking potential or in combination with dairy proteins need more physical and physicochemical characterisation, for testing their ability to act as FRs after suitable processing, which needs future research attention.

## 3 Protein-based microparticles as fat replacers

**Fig. 2:**
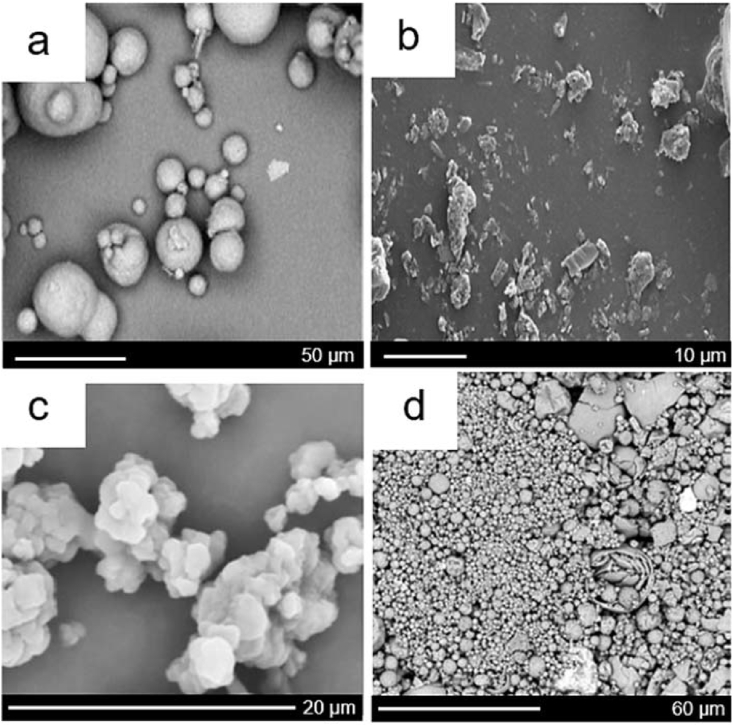
Scanning electron micrographs (SEM) of microparticulated proteins; (a) microparticulated whey protein (MWP), (Liu, Tian, Stieger, van der Linden, & van de Velde, 2016c), (b) microparticulated egg white protein MEWP) (Liu et al., 2018a), (c) microparticulated soy protein (MSP) (Zhang et al., 2020b) and (d) microparticulated hemp protein (MHP) (Beran et al., 2018). Micrographs are reproduced with permission from Elsevier (a, c), J-STAGE (free access) (b) and Longdom Publishing SL (open access) (d).

One of the most extensively investigated research areas for fat replacement has been the use of microparticulated proteins as FMs. Microparticulated proteins (Fig. 2) are mostly dried protein particles which are of smaller particle size compared to concentrate/isolate protein (0.1–20 μm diameter) that are created using thermal treatments and high shear processes at low pH (Ipsen, 2017). Such microparticulation processes came to existence through the first patent in 1988 on whey protein, which was launched with a commercial name of Simplesse® (Singer, Yamamoto, & Latella, 1988) by NutraSweet. The patented microparticulated protein by this thermo-mechanical process claimed to have “a substantially smooth, emulsion-like organoleptic character when hydrated”. This was followed by a second patent two years later covering microparticulated bovine serum albumin (BSA), egg white protein (EWP) and plant proteins, such as soy protein (Singer, Latella, & Shoji, 1990). Once the patent expired, other commercially available microparticulated proteins surfaced with brand names such as APV LeanCreme™ (SPX technology) (Rajakari & Myllarinen, 2016) or other containing combinations of microparticulated protein with polysaccharides or carbohydrates, such as Dairy-Lo® (valentino & baughman, 2004) containing microparticulated protein + microparticulated cellulose). The key feature of microparticulated proteins is that they mimic the spherical shape and size of emulsified fat droplets (Fig. 2) and are postulated to create a creamy mouthfeel by ‘ball-bearing’ mechanism (Cheftel & Dumay, 1993; Liu, Tian, Stieger, van der Linden, & van de Velde, 2016c). Few non-dairy microparticulated proteins such as zein, egg and plant protein exist and have been used as FRs. Recently, microparticulated proteins from such alternative sources have started populating the FR landscape with egg white protein (MEWP) being used in salad dressing (Liu et al., 2018a), microparticulated plant proteins in gluten-free bread (Beran et al., 2018), in yoghurt (Dabija et al., 2018) and model systems (Zhang et al., 2020b). Here, we discuss the applied research performed using microparticulated whey protein, microparticulated forms of egg and plant proteins in different model and real food applications that have been investigated in literature in the last 5 years using a range of rheological and tribological techniques (Table 2).

**Table 2:** A summary of micro-particulated proteins incorporated as fat replacer in a range of applications in the past 5 years of research.

Fat Replacers (FRs)		Application	Particle size (μm)	Production conditions	Key findings	References
Animal protein-based FRs						
MWP/NWP		Model gels	0.1–1	- 0–8% w/w MWP - Measured at 20 °C	↑ η ↓ μ	(Liu, Tian, Stieger, van der Linden, & van de Velde, 2016b)
			0.2	- 0.6-4.4% w/w MWP -HTST treatment - pH 6.6 (milk gel pH 4.8) -Measured at 4 °C	↓ η ↓ G’ ↓yield stress ↓tortuosity ~ Cohesiveness ↓ H_2_O capacity ↓ firmness ↑ porosity	Silva and O'Mahony (2018)
		Skimmed milk	1.16	- 0.5–20.0% w/w MWP - Measured at 25 °C and 37 °C	↑ η ↓ μ ↓ n	Olivares et al. (2019)
		Stirred yoghurt	0.005–100	- 4.25% and 5.0% w/w (total protein) MWP, NWP, 10 variations - Pasteurised - Yoghurt pH 4.6 - Measured at 4 °C	↑ η (high N/D) ~ η (low N/D) ↑ Gel strength ↑ G’ (high N/D) ↑ Elasticity ↑compaction ↑ H_2_O capacity (high N/D) ↑ Mechanical resistance (high N/D)	Torres et al. (2018)
		LF Edam cheese	–	- 0.3–0.9% w/w MWP, 3 variations. - Pasteurised - 4.5–5.3% salt (in moisture) - Measured at room temperature	↑ moisture, ↑ proteolysis, ↓ firmness, ↑ homogeneity ↑ texture sensory: ↓ texture (highest Simplesse®100, Prolo®11), ↑ flavour, ↑ appearance ↑ OA ↑ bitterness (highest Simplesse 100, Prolo®11)	El-Aidie et al. (2019)
		LF cheese emulsion	0.01–3	- 5–20% (of dry matter, 16–28%) MWP - Heated at 80 °C and sheared - pH 4.7–4.9 - 3% w/w salt Measured at: 45 °C	↑ followability ↓ η ↑↓ n ↓ shear stress ↑ porosity ↑ Whiteness; Sensory: ↑ flowability ↑ Glossiness ↓ Mouth-coating	Urgu et al. (2019)
		LF Pickled cheese	–	- 1% w/w MWP - Pasteurised - pH 5.1–5.2 - 2.5–3.5% w/w salt - Measured at 4 °C	↑ moisture ↓ hardness ↑ springiness ↓ cohesiveness ↓ gumminess ↓ chewiness, Sensory: ↑ whiteness ↑ appearance ↑ OA ↑ odour ↑ flavour	Akin and Kirmaci (2015)
		Processed cheese	0.01–3	- 3–7% w/w MWP - Heated 90 °C 5 min - pH 5.6 - Measured at 5 °C	↑ G′ ↑ G″ ↓ Max tan *δ* ↑ Gel-sol transition temp ~ firmness ~ hardness ~ colour	Schädle et al. (2020)
		Kefir	–	- 2% w/w MWP, 2 variations - Heated 93 °C 15 min - pH 4.3–4.4. - Measured at 20 °C	↑ η, Sensory: ↑OA	Temiz and Kezer (2015)
		Multi grain cookies	–	- 7% w/w MWP - 0.2% w/w salt - Heated 165 °C 16 min - Measured at room temperature	~ colour ~ flavour ↓ OA ↓ Tenderness	Aggarwal et al. (2016)
EWP		Salad dressings	9.42	- 9–11 g/mL MEWP sample vs commercial salad cream - Heating 13 min, sheared - pH 3.6	↑ η ↑ ↓ n ↑ G’ ↑ G’’ ↑ tan(*δ*) ↓ thixotropy; Sensory: cohesiveness **~** appearance ↓ flavour ~ OA	(Liu et al., 2018a)
Plant protein-based FRs						
Micro-particulated plant proteins	MSP, MSP + EWP	Model liquid	5.0–20.0	- 6–15% wt/wt MSPI, MSPI + EWP - Heated 95 °C 30 min - pH 7 - Measured at 25 °C	↓ μ (MSPI), ↓↓ μ (EWP + MSPI) ↑ η↓ off-flavour volatiles	Zhang et al., 2020b

### 3.1 Microparticulated whey proteins

Simplesse®, the first microparticulated whey protein (MWP) available commercially was created by thermal aggregation and intense shearing at low pH (pH 4.0–5.5) resulting in the thermal denaturation of *β*-lg, *α*-la and BSA, whereby the thiol groups of *β*-lg are exposed for covalent disulphide interaction. Lower electrostatic repulsive forces using relevant pH enabled hydrophobic interactions in the proteins to take place resulting in aggregation and finally forming particles via shearing process with particle size of <5 μm (Beran et al., 2018; Liu et al., 2018a; Toro-Sierra, Schumann, & Kulozik, 2013). As can be seen from micrographs, MWP is spherical and smooth with particles having no sharp facets (Fig. 2a), however, there are also reports of development of large coarse aggregated particles of >30 μm size during the spray drying process. Spray drying has been shown to affect particle size and native/denatured (N/D) ratio in whey proteins. Torres, Janhøj, Mikkelsen, and Ipsen (2011) investigated ten MWP types produced using different processing methods and found that a high N/D whey protein ratio (0.94–1.33) in the MWPs generated LF yoghurt with enhanced viscosity, yield stress, storage modulus (G’) and creaminess perception (Table 2). In contrast, another study (Toro-Sierra et al., 2013) found that 90% of protein was found to be of the same size before and after drying with little significance of inlet and outlet temperature, suggesting controlling temperature alone was not sufficient to attain a certain particle size.

In LF dairy foods, MWP has shown ability to improve organoleptic (creaminess, lubrication) and functional (viscosity, flow) properties (Ipsen, 2017, Liu et al., 2016a). In addition to dairy matrices, MWP has also been successfully used in mayonnaise (Sun et al., 2018) and glazes (Meza, Peralta, & Zorrilla, 2016). However, use of MWP as FRs in baked foods such as cookies (Aggarwal, Sabikhi, & Sathish Kumar, 2016) and fried products (Pokorny, 1998) was not promising owing to the high temperature conditions used in these processed food, leading to thermal interaction of MWP with other ingredients resulting in loss of fat like texture (Lucca & Tepper, 1994; O'Connor & O'Brien, 2016). Furthermore, MWP has not shown success in ice cream when used as a sole FR as it lowered viscosity, smoothness and mouth coating and increased generation of undesirable volatiles (Welty, Marshall, Grün, & Ellersieck, 2001). However, in combination with polysaccharides, MWP has shown synergistic effects to replace fat in ice cream (Ipsen, 2017). Considering a detailed review already exists on applications of MWP in dairy applications (Ipsen, 2017), we have only focussed on research carried out after 2017 in this application area unless there is a mechanistic information that applies to other applications.

**Model systems.** Liu, Tian, Stieger, van der Linden, & van de Velde, 2016c postulated for a ball-bearing type mechanism of lubrication whereby small spherical MWP particles (less than 5 μm in diameter (see Fig. 2a) are able to roll across the surfaces, reducing friction, proposing to mimic emulsified-fat droplet inducing a creaminess-like sensation in the mouth. In the presence of small quantities of MWP, μ was greatly reduced but at ≥3 wt% MWP, the lubrication effect was significantly reduced with plateau between 5 and 8%, likely because of saturation of these particles between the tribo-pair surfaces preventing the rolling mechanism to persist (Table 2). This ball bearing effect was also observed to be better in liquids than in semi solids. In a recent study, friction behaviours similar to FF emulsions (0.5–20.0% w/w fat) were again demonstrated using various concentrations of MWP (Simplesse®) at sliding speeds (10–50 mm s^−1^) typical of oral processing (Olivares et al., 2019). Size is known to be vital where small particles of MWP were associated with body and richness but sizes above 10 μm diameter were detected with roughness, and increase in μ values. However, the viscosity and perceived thickness of the medium was found to be effective in masking the larger particulates (Liu, Stieger, van der Linden, & van de Velde, 2016b, Liu, Tian, Stieger, van der Linden, & van de Velde, 2016c) . From this study it was concluded, MWP can help to reduce rather than replace fat in food as MWP did not replicate the fattiness in sensory, due to lack of any oily film formation, thus, limiting usage of MWP for FF replacement.

**Milk gels.** In high protein acid milk gels, MWP has been shown to interact with casein, forming a more open structure creating a softer product compared to control *i.e.* milk gels containing no added MWP (Silva & O'Mahony, 2018). MWP gels were characterised as softer, lower firmness, consistency and cohesiveness with significantly increased porosity as well as a decrease in tortuosity as a result of the open structure. Addition of nanoparticulated whey protein (NWP) (particle size of <1 μm) resulted in higher firmness of the acidified gels through both covalent and non-covalent interactions (Table 2).

**Yoghurt.** In yoghurt, removal of fat leads to increased syneresis, weaker body and unsatisfying texture. Addition of MWP (Torres et al., 2018) has been shown to reduce such detrimental characteristics. For instance, in LF yoghurt containing MWP having higher ratios of native *β*-lg/*α*-la in the MWP resulted in higher elastic modulus, better flow behaviour (*n*), lower syneresis, dense aggregates and low serum pores (observed using scanning electron microscopy, SEM) in LF yoghurt closely resembling FF yoghurt (Torres et al., 2018). The small particle size of MWP, such as the use of NWP in yoghurt, might have behaved as ‘active fillers’, which might have further increased the viscosity and *G’* (Torres, Murray, & Sarkar, 2016) of LF yoghurt. Nevertheless, MWP has also been reported to act instead as ‘inactive fillers’ not associating with the gel network (Sandoval-Castilla, Lobato-Calleros, Aguirre-Mandujano, & Vernon-Carter, 2004). In such cases, electron micrographs have shown LF yoghurt with a more open network with localised dense areas of protein, which may result in a lower WHC. Furthermore, where MWP are larger than that of fat globules, it creates a higher level of serum separation, increased graininess and lower firmness as compared to whey protein concentrate (Table 2).

**Cheese.** In case of cheese, fat is integral to structure, making up approximately 20–30% of the product. Although, commercially available LF cheeses can be distinguishable from FF cheeses based on tribological and sensory properties (Laguna et al., 2017), the incorporation of MWP in cheese can be beneficial in terms of sensory attributes. In LF Edam cheese, the addition of three types of commercially available microparticulated whey protein (Simplesse®, DairyLo® and Protelo®) at 0.3–0.9% was demonstrated to reduce firmness, restore proteolysis, opaqueness and improve sensory acceptability (El-Aidie et al., 2019). The MWP was shown to act as an ‘inactive fillers’, as opposed to active ones as seen in yoghurt (Torres et al., 2018). Addition of Simplesse® at 0.9% level significantly opened up the microstructure resulting in the lowest firmness, this effect was also seen with another study of LF pickled cheese with Simplesse® at 1% (Akin & Kirmaci, 2015) however a study using higher concentrations (3–7% w/w) found that hardness and firmness increased which limits the effective use of MWP in cheese to small concentrations (Schädle, Eisner, & Bader-Mittermaier, 2020) (Table 2). In terms of sensory properties, the water retention properties of the MWP that is proportional to firmness of the cheese correlated directly to sensory appreciation of optimum body and texture. It was found that all three MWP at 0.6% resulted in optimum body and texture with DairyLo® and Protelo® with better flavour than Simplesse® (El-Aidie et al., 2019) (Table 2). In the case of pickled cheese, LF cheese with Simplesse® achieved better sensory results in all aspects as compared to the FF cheese.

In case of spray-dried cheese powders, removal of fat from the liquid pre-emulsified cheese leads to an 14 × increase in viscosity, resulting in severe issues with atomisation as well as detrimental sensory changes in the finished cheese powder (Urgu et al., 2019). Addition of 3% MWP (in dry matter of 20%) has shown to create similar open porous structures as that of FF cheese powders restoring the original viscosity and increased lubrication, improving flow rate in piped systems. Furthermore, MWP enhanced glossiness and were able to replicate mouth coating scores of FF cheese powders at 3% with no off flavours detected even at highest concentration of MWP (4%) (Urgu et al., 2019). However, interestingly increasing MWP in the LF cheese lead to proportional decreases in mouth coating and at high dry matter (the optimum 25% in FF cheese emulsion), MWP was not able to replicate instrumental viscosity or sensory thickness suggesting that a change in processing and formulation design might be required when using MWP as FR.

**Non-dairy foods.** Most applied research on MWP has been conducted in dairy applications with rare attention being given to non-dairy systems. For instance, in multigrain cookies MWP failed to significantly perform as a FR, the cookies received the lowest sensory, texture, colour and overall acceptability score (Aggarwal et al., 2016) (Table 2). The cookies became tender in texture and darker in colour due to the maillard reaction of excess amino acids from the MWP with the reducing sugars. However, MWP showed promising effects in replacement of fat in glazes (Meza et al., 2016). Flow properties of glazes made of high sugar were replicated with replacement of sunflower oil with Simplesse®. Both 6% fat (in FF glazes) and 9% MWP (in NF glazes) increased yield stress, viscosity, consistency coefficient (*K*), viscoelasticity and film thickness with no significant differences between the FF and LF glazes (Meza et al., 2016). MWP was able to bind excess water and restricted metastable sugar transformation into larger and harder crystals. It was revealed microscopically that NF glazes with 6% MWP and FF glazes with 9% fat had similar sized sugar crystals and no discontinuities in the crystal matrix, in contrast with no fat, no MWP glazes.

Ultimately MWP has an extensive history of use as a FR and it has dominated the FR domain with application to foods, however the limitations of the level of fat being replaced vary depending upon the size of MWP used and viscosity of the continuous medium. In addition, emerging research attention is now paid to alternative proteins and new methodologies to advance the understanding of fat replacement which is discussed later.

### 3.2 Microparticulated egg white protein

Besides microparticulation of whey protein, microparticulated egg white protein (MEWP) has also been reported in literature where applied research may have been driven by growing consumer demands for LF mayonnaises, salad dressing *etc.*, where egg is a key ingredient. As can be observed using the SEM image in Fig. 2b, MEWP is non-spherical in shape with mean size of 9.42 μm (Liu et al., 2018a) and with sharp facets unlike the smooth spherical structures of MWP (Fig. 2b). Therefore, it remains uncertain if MEWP could roll and lubricate via the ‘ball bearing mechanism’ as proposed for MWP. Nevertheless, in this study the overall acceptability of MEWP salad dressings was demonstrated to be comparable to that of commercial salad dressing at 35% fat (Table 2). MEWP has been scarcely investigated in literature and so further research using MEWP in various food applications and exploring rheological and especially tribological properties of non-spherical MEWP/other proteins, could be a topic of future consideration.

### 3.3 Microparticulated plant proteins

Plant protein-based microparticles have only recently been considered as FRs with very limited studies to date. Soy protein isolate has very recently been microparticulated (MSP) by heating a 15% dispersion at 95 °C (5–15 min), homogenising and spray drying (Zhang et al., 2020b). As shown in Fig. 2c, MSP displays highly irregular non-spherical particle clusters with a mean size of 2–20 μm. Interestingly, MSP (10% w/w protein dispersion) showed an effective reduction in friction coefficient (μ ~ 0.1) at orally relevant speeds of 50 mm/s (Zhang et al., 2020b), similar to what was obtained with 20% MWP in milk (Olivares et al., 2019). The high rigid particles enhanced by increased disulphide bonds were thought to be reasons for lower friction, although irregular in shape, which may suggest as with MEWP surface topography may not be vital for lubrication. Recently, hemp and canola protein extracted from filter cakes have also been microparticulated to create microparticulated hemp protein (MHP) and microparticulated canola protein (MCP) (Beran et al., 2018). Microparticulation was performed using carbon-dioxide nebulisation, combining drying and micronisation at lower temperatures (25–65 °C) with saturation of carbon-dioxide at 4–8 MPa, the resulting powder was then separated using a cyclone to produce nano-sized particles with enhanced solubility and functionality. In comparison to MWP, MHP had an open hollow sphere-type of architecture, with surface cracks and had high degree of polydispersity as can be seen in Fig. 2d. Interestingly, MHP has an average size of 0.6 μm, smaller than that of Simplesse® (1 μm). This unique production of plant microparticles offer potential for fat replacement and needs further research attention in application for other food. However, one should not overlook that MHP and MCP suffered from astringency issues when applied in baked goods (Beran et al., 2018), which might be associated with lubrication failure phenomena limiting FR use.

Dual protein microparticulation is a relatively recent endeavour for improving the functionalities of plant protein-based microparticles. In this direction, recently SPI and EWP were elegantly co-microparticulated (4:1 SPI/EWP) by Zhang et al., 2020b (Table 2), where EWP acted as a thiol group donor. Such combination with EWP led to better protein-protein gelation due to disulphide crosslinking resulting in final microparticles (M (SPI + EWP)) with much smoother surface as compared to the MSP shown in Fig. 2c. The remarkable feature of this new microparticulated co-protein was that it enabled friction coefficient reduction by an order of magnitude from 0.1 to 0.01 at orally relevant speeds of 50 mm/s (T. Zhang et al., 2020b) highlighting synergy of using these two proteins for lubrication performance. However, authors reported that the viscosity of this co-protein was greatly reduced by over 75% compared to standard MSPI (12–15 wt%) and hence, may have a softening effect in final food application.

Overall there has been little research in plant/alternative proteins and needs much focused attention. It is unclear how much fat can be replaced using plants protein/co-proteins, but tribological evidence offers early promises that it may promote fatty like behaviours in LF foods. Furthermore the unique structural, physiochemical and mechanical properties of plant proteins as well as such vast varieties of plant sources offer strong future development for a number of fat replacement uses using plants in our food.

## 4 New protein-based microgels – as future fat replacers

**Fig. 3:**
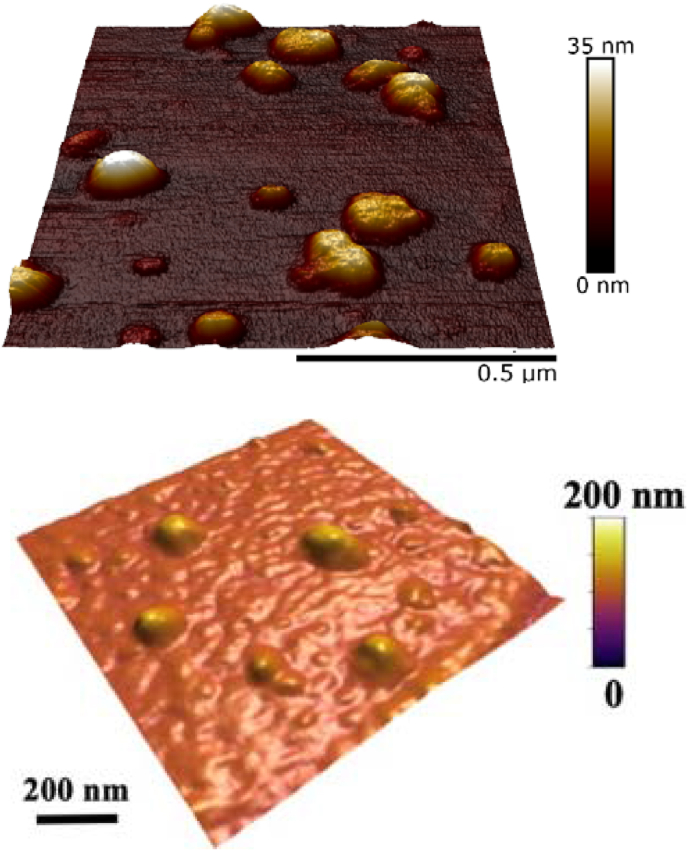
Atomic force microscopy (AFM) images of whey protein microgel particles obtained by a (a) top down (Andablo-Reyes et al., 2019), reproduced with permission from RSC (open access) or (b) bottom approaches (Bahri et al., 2019), reproduced with permission from Elsevier.

In recent years there has been much interest in the development of protein-based microgels, which shows strong potential to act as future FRs. These protein-based microgels are soft colloidal particles that are produced by preparing physically cross-linked heat-set hydrogel (90–95 °C, 10–30 min) using highly concentrated protein solutions, followed by breaking them down to micrometer-or nanometric-sized gel particles under high shear forces. A combination of steric and electrostatic repulsions confer good colloidal stability to these particles in aqueous dispersions (Dickinson, 2017). In our laboratory, protein microgels have been synthesized using the above-mentioned top down approach from highly concentrated whey protein (Araiza-Calahorra & Sarkar, 2019; Sarkar et al., 2016), pea protein (Zhang, Holmes, Ettelaie, & Sarkar, 2020a), egg white protein (Li, Murray, Yang, & Sarkar, 2020) or lactoferrin (Sarkar et al., 2018) that is gelled via thermal aggregation and subsequently homogenized to create microgels or even sub-micron sized nanogels ranging in size from 80 to 90 nm (see Fig. 3a) (Andablo-Reyes et al., 2019; Araiza-Calahorra & Sarkar, 2019) to 300–400 nm (Sarkar, Kanti, Gulotta, Murray, & Zhang, 2017).

Protein microgels can also be made by cold gelation of denatured proteins using calcium ions followed by shearing process (Ni et al., 2015; Torres et al., 2017), gelation via enzymatic approaches followed by shearing (Jiao, Shi, Wang, & Binks, 2018) or by bottom up approaches involving pH- and temperature-induced aggregation of proteins into microgels (see Fig. 3b). Microgels fundamentally differ from microparticulated proteins as the former is swollen and saturated with water providing unique rheological capabilities that lie in between the protein and the gel. Processing techniques have been well-documented in literature to control vital characteristic such as particle size and elastic modulus of the microgels. For instance, it was found that a ten-fold decrease in particle size can be obtained by using lower concentration of calcium ions and lower turbulent mixing time in case of cold gelation technique (Torres et al., 2017). Protein microgels also undergo pH-dependant alternation in their microstructure and deformability. For instance, a 15-fold increase in stiffness was reported at pH 5.5 and 3.0 in whey protein microgels as compared to being at neutral pH (Bahri et al., 2019).

Generally, microgels have a shear-thinning character depending upon the volume fractions of the microgels and have been recently proven to act as excellent bio-lubricants. They tend to reduce friction either acting as physical surface separators between tribo-contact surfaces or the gel particles roll via ‘ball bearing mechanisms’, latter is often postulated owing to the smooth, spherical nature of these swollen soft particles as can be observed in the atomic force microscopy images (Fig. 3) (Andablo-Reyes et al., 2019; Bahri et al., 2019). Of the few reports available on tribology in whey protein microgels (WPM), increasing volume fractions 10–80% has been demonstrated to lead to decrease μ by a factor of 10 in hydrophobic tribological surfaces (Sarkar et al., 2017). Interestingly, ‘soft’ WPM (G’ ~ 100 Pa, prepared with 10 wt% WPI) was found to be less lubricating than ‘hard’ WPM (G’ ~10 kPa prepared with 15 wt% WPI) (Andablo-Reyes et al., 2019). For example, a two fold increase in viscosity was recorded with hard microgels with enhanced lubricating capabilities. Such reduction in μ was also seen when the WPM was dispersed in a continuum ranging from buffer to other Newtonian (corn syrup) and non-Newtonian mediums (xanthan gum) depending upon the stiffness of the WPM (hard versus soft) as well as viscosity and rheological behaviour of the continuum (Andablo-Reyes et al., 2019), which may have beneficial application when designing LF products.

Currently, microgels are still being physicochemically characterised in literature and in present times, there exist a number of hindrances for its commercial use as a food ingredient. One is the control of microgels swelling/deswelling ability in response to environmental pH, ions and temperature. Changes to the environment as well as interaction with other ingredients may create stability issues in the microgel during storage and processing. The processing of microgels is complex and particles are often irregular in morphology, conditions would need to be strictly controlled and further clarification of unwanted polymer by-products (*i.e.* non-microgelled protein in the continuous phase) would make it an overall expensive processing technology. Microgels contain >90 wt% water and economically should be transported in a dry state, commonly cost-effective drying techniques (*i.e.* spray drying) may impact the morphology and functionality of the microgels. Literature has shown evidence that freeze drying is effective with little physicochemical changes upon rehydration, however, such data is limited to non-food grade poly(*N*-isopropylacrylamide) microgels (Agbugba, Hendriksen, Chowdhry, & Snowden, 1998) and food grade application is limited to soy protein microgels (Benetti, Silva, & Nicoletti, 2019).

In summary a paucity of tribology data in microgels other than WPI and no sensory data in microgels to date suggests further research is required in this domain. Considering the viscosity modifying properties and high lubricating ability, microgels offer strong candidacy as FRs, which needs to be investigated particularly from sensory viewpoint in model foods primarily, followed by its application in real food systems and cost-effective upscaling of microgels needs to be explored. One should also consider investigating the properties of these swollen microgels versus hydrated versions of dried microgels to really understand whether the microgels get swollen to the optimized state after drying.

## 5 Summary of design features for protein-based fat replacers

**Fig. 4:**
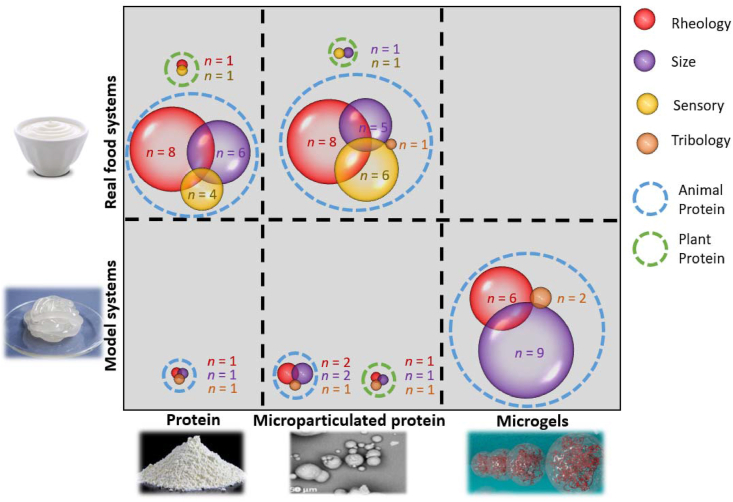
Bubble plot illustrating the characterisation techniques, protein types used as fat replacers including microgels with applications in real or model systems discussed in the review, where n = number of studies (see Supplementary Table S1) for raw data.

To summarize the overall landscape of FRs in last 5 year and clearly identify the knowledge gap, Fig. 4 illustrates the characterisation techniques, number of studies, the systems and forms of the proteins used in model and real food systems that have appeared in literature in the last half a decade. In model systems, there are relatively few studies using protein concentrates/isolates and microparticulated proteins as FRs with a more saturated research effort in case of protein microgels. It is well recognized that model systems are ideal for fundamental understanding and generating mechanistic insights on how proteins can act as FRs. Therefore, the limited research in understanding the role of proteins and microparticulated proteins in model foods in last 5 years might be attributed to the extensive work already done in the past (Lucca & Tepper, 1994; Nateghi et al., 2012) with the level of research slowing down. This is in close agreement with significant levels of research done in real foods with proteins and microparticulated proteins. This suggests that research in use of proteins and microparticulated proteins have progressed well over the last few decades where the paradigm has been shifted towards research in real food applications. In stark contrast, microgels have been employed only with model systems to gain fundamental understanding as they have been synthesized at laboratory scale. To our knowledge, no paper has reported using microgels as FRs in real food application microgels. Nevertheless, a patent search shown in Table 3 shows examples of proteinaceous microgels or microgel-like particles that have been applied in fat replacement restricted to mostly diary with some condiment and confectionary applications. Advancements in microgel preparation, appropriate scale up, characterisation of their lubrication capabilities and sensorial properties may offer wider application of microgels in real food use and consequently will advance the field. This clearly highlights an untapped opportunity space for exploration by researchers and food industries in the future. In regards to protein type, there are only few studies using proteins from plant sources in all areas of fat replacement presenting further opportunity considering the global rise of sustainability demands and rapid rise in microparticulated plant proteins as discussed previously.

**Table 3:** List (non-exhaustive) of patents using microgels or microgel-like particles for fat replacement applications.

Patent name	Reference	Filing date	Current Assignee	Size	Examples of fat replacement applications
Protein product	EP0323529B1 (Singer et al., 1987)	1987	Labatt Breving Co Ltd	0.1–2.0 μm	Ice cream, mayonnaise, salad dressing
Insoluble protein particles	EP1292196B1 (Villagran et al., 2001)	2001	The Folgers Coffee Company	<2 μm (preferentially 0.1–3.0 μm)	Ready to drink beverages
Low fat creamer compositions	EP1589821B1 (Villagran and Baughman, 2003)	2003	Procter and Gamble Co	0.1–10.0 μm (preferentially 0.1–5.0 μm)	Coffee and tea creamer
Satiety enhancing food product and a method for manufacturing such	EP1833309A1 (Adams et al., 2005)	2005	Unilever PLC Unilever NV	<300 μm	Meal replacer
Whey protein micelles	EP1839492B1 (Schmitt et al., 2006)	2006	Nestec SA	<1 μm (preferentially 0.2–0.4 μm)	Dairy desserts, coffee creamers, fat based condiments, chocolate, spreads
Creamer composition comprising plant protein microparticles	EP2991498A1 (Schmitt and Rade-Kukic, 2014)	2014	Nestec SA	100–4000 μm	Coffee and tea creamer

In both real food and model food systems (Fig. 4), rheology and particle size measurement appear to dominate the characterisation space for fat replacement as these have been postulated to have strong mechanistic and empirical association to some of the sensory attributes (Foegeding, 2007). In contrast to model food systems, sensory characterisation is very popular in real food systems, which is definitely the most important test to identify whether or not the NF/LF products using the FRs can replicate the sensory perception of FF products. It is notable that sensory analyses are limited in model foods, this is largely associated with difficulty in training participants with model food systems as compared to trained panellists recruited in food industries for real food systems. Oral tribology is now acknowledged as a promising tool to relate to mouthfeel perception that occur in the later stages of oral processing and cannot be explained by rheological analysis alone (Pradal and Stokes, 2016; Sarkar, 2019; Sarkar and Krop, 2019). A lack of appreciation of tribology in real food systems is apparent (Fig. 4) as no studies have yet been performed. Recent studies on model proteins, microparticulated proteins and microgels have incorporated tribology given its importance in understanding the role of fat and protein-based FRs in lubrication. Especially with advancements of fundamental tribological studies and tribology-sensory correlations, it should be a key area of characterisation in future research when designing FRs or testing FRs in model and real foods for designing NF/LF products. It is also important to highlight that recent advancement of promising adsorption techniques such as quartz crystal microbalance with dissipation (QCM-D) using saliva-coated surfaces may complement the tribology analysis (Xu et al., 2020). Such combination of tribology with muco-adhesion experiments may be a powerful approach in the understanding of the interactions of FRs with saliva and thus help to reduce the development cycle of food product development by reducing the number of sensory trials.

We postulate in the following some key take-home messages on physiochemical and structural properties for designing protein-based FRs in order to be functionally viable for designing LF/NF products with appropriate sensory perception:

**Particle size, shape and rigidity.** Particle sizes in protein-based FRs range from aggregated protein isolates at 46–168 μm, microparticluation proteins (0.2–9 μm) to sub-micron sized (0.04–0.3 μm) particles in case of microgels. It is evident from literature that small particle size is important for boundary lubrication to allow entrainment of the particles in the narrow gap between the tongue and palate surfaces during oral processing (or tribopairs in oral tribology experiments), with particle sizes greater than 10 μm often being perceived as gritty and with increased friction. Particle shape may be important, where irregular-shaped particles with sharp facets could cause reduced creaminess perception where-by spherical particles offer ‘ball-bearing’ lubrication mechanism, but needs further investigation. Besides size and shape of the microparticulated proteins and microgels, rigidity of particles are very important and can influence the mouthfeel perception by virtue of their tribological properties (Andablo-Reyes et al., 2019; Araiza-Calahorra & Sarkar, 2019). Therefore, modifying size and shape of FRs with appropriate deformability to closely resemble fat-based emulsion droplets might be an appropriate approach to generate mouthfeel in LF/NF products similar to that of FF products.

**Rheology.** All FRs have shown the ability to greatly modify the bulk viscosity in both model and real food systems. Fat has viscosity enhancing properties, as such, LF foods tend to suffer from 25 to 50% decrease in this attribute both measured using instrumental and sensory techniques. Most FRs are therefore designed so that they can contribute to water binding ability. Another important feature is that many, if not, most fat-based emulsions have a shear-thinning behaviour. Of course such properties are difficult to achieve using proteins on its own as aqueous dispersions of protein tend to have a Newtonian behaviour. Hence, proteins combined with polysaccharides (xanthan gum, cellulose) are often used to improve the viscosity of the food matrix. In addition, microgels can be particularly interesting as FRs as they possess excellent shear thinning behaviour in orally relevant shear-rates (Andablo-Reyes et al., 2019; Araiza-Calahorra & Sarkar, 2019), however such rheological behaviour might change depending upon the food matrix as well as rigidity of the particles (Andablo-Reyes et al., 2019; Araiza-Calahorra & Sarkar, 2019), which needs to be investigated in the future.

**Tribology.** Fat acts as a lubricant film and thus has the ability to reduce the friction coefficient between the tongue and oral palate in the later stages of oral processing where the thickness of those fatty films range from few molecules to hundreds of nanometres of thickness. Recent work with proteins, microparticulated proteins and microgels have shown lubrication attribute with specific attention to ball bearing mechanism. However, often, there is lack of direct evidence for the proposed underlying mechanism. Hence, it is crucial to understand the in situ flow-induced structural alterations of microparticulated proteins and microgels under shear. For instance, it might be interesting to combine tribological set up with small angle neutron-scattering facility to reveal the structure of the lubricating film between the tribo-pair while it is sliding. Also, lubrication behaviour of microgels and microparticulated systems is also dictated by size, shape, rigidity and viscosity, which needs focused attention. In general, the FRs that can reduce boundary friction to a large extent and when introduced in LF/NF products provide comparable friction coefficient to that of FF products should be a useful tool to screen FRs. In general, creaminess is a highly complex mouthfeel perception that is mostly present in FF products and not in LF/NF products. Generally, creaminess is correlated to smoothness, fatty mouth feel and creamy flavour (Frøst & Janhøj, 2007), which can be predicted using combination of complementary assessment of particle size, shape, rheology, tribology and QCM-D.

**Protein-ingredient interactions** Proteins are highly responsive to other ingredients and environments (*i.e.* heat, pH, ions) which may cause challenges when used as a FM. Proteins vary in charge across pH ranges and the net charge is influenced by the ionic conditions leading to variations in protein-protein and protein-ingredient interactions, which may result in macroscopic physical and chemical changes. Fundamentally the water binding ability of proteins may limit availability of water for other components, leading to rheological and microstructural implications. It is well known proteins interact with various thickeners used in food applications such as carrageenan (Drohan, Tziboula, McNulty, & Horne, 1997). Such interactions may be repulsive or attractive which may govern phase macroscopic separation (Goh et al., 2008). Heated foods presents an additional degree of challenge for proteins as FR. At prolonged high temperatures, typical of baked foods, aggregation and loss of water binding is expected resulting in macro-structural changes. Above 140 °C, the available amino acids in protein-based FRs and reactive carbonyl from reducing sugar in the food *i.e.* monosaccharides, lactose, maltose may react causing browning and generation of complex flavour compounds, known as the maillard reaction, which might hinder applications of protein-based FRs in baked foods.

## 6 Conclusions

In order to design low fat/no fat products that mimic the behaviour of full fat products, there has been extensive research in last few decades using proteins and microparticulated proteins. Whey protein appears to dominate the field with most usage in dairy fat replacement. There has been recent interests in the use of plant proteins, however characterisation, understanding and application is highly limited to date. Besides particle size, rheology and sensory evaluation, tribology appears to be a new tool that is being applied to understand the lubrication properties of fat replacers mostly in model food systems. Combination of tribology with muco-adhesion techniques can be powerful screening tools for identifying fat replacers with just-right mouth feel properties in the future for sensory testing. Besides proteins and commercially available microparticulated proteins, microgels that are designed in laboratory-setting have recently demonstrated superior rheological and lubrication performance with ability to act as potential fat replacers. However, information lacking in sensory perception of microgels in literature and the challenges of tailored commercial scale microgel production appear to be the bottlenecks to find its usage in real food applications.
