# Supplementary material for: Review on fat replacement using protein-based microparticulated powders or microgels: A textural perspective
Source: Trends Food Sci Technol. 2020 Dec;106:457–68. doi: 10.1016/j.tifs.2020.10.032 (PMC7763486; doi:10.1016/j.tifs.2020.10.032)
Supplement: Multimedia component 1 [file mmc1.docx]

**Supplementary Document**

**Review on fat replacement using protein-based microparticulated powders or microgels: a textural perspective**

Ben Kew^1^, Melvin Holmes^1^, Markus Stieger^2^ and Anwesha Sarkar^1^*

^1^Food Colloids and Bioprocessing Group, School of Food Science and Nutrition, Faculty of Environment, University of Leeds, Leeds, LS2 9JT, UK

^2^Division of Human Nutrition and Health, Wageningen University, PO Box 17, 6700 AA Wageningen, The Netherlands

Corresponding author:

*Dr. Anwesha Sarkar

Food Colloids and Bioprocessing Group,

School of Food Science and Nutrition,

University of Leeds, Leeds LS2 9JT, UK.

# **Supplementary Table S1.** Characterization techniques used in various studies involving protein—based fat replacers and microgels.

| Protein types | References | Size | Rheology | Sensory | Tribology |
| --- | --- | --- | --- | --- | --- |
| Protein Concentrates/isolates | Zhang, 2015 | ✓ | ✓ |  |  |
|  | Costa, 2016 |  | ✓ |  |  |
|  | Fang, 2019 | ✓ | ✓ | ✓ |  |
|  | Jørgensen, 2015 | ✓ | ✓ | ✓ |  |
|  | Guo, 2018 | ✓ | ✓ | ✓ |  |
|  | Liu, 2018 | ✓ | ✓ | ✓ |  |
|  | Zhu, 2019 | ✓ | ✓ |  | ✓ |
|  | Dabija, 2018 |  | ✓ | ✓ |  |
|  | Danesh, 2018 | ✓ | ✓ |  |  |
|  | Schädle, 2020 |  | ✓ |  |  |
| Micro-particulated protein | Aggarwal, 2016 |  |  | ✓ |  |
|  | El-Aidie, 2019 | ✓ | ✓ | ✓ |  |
|  | Torres, 2018 | ✓ | ✓ |  |  |
|  | Temiz, 2015 |  | ✓ | ✓ |  |
|  | Akin, 2015 |  | ✓ | ✓ |  |
|  | Urgu, 2019 | ✓ | ✓ | ✓ |  |
|  | Olivares, 2019 | ✓ | ✓ |  | ✓ |
|  | Liu, 2018 | ✓ | ✓ | ✓ |  |
|  | Beran, 2018 | ✓ |  | ✓ |  |
|  | Zhang, 2020a | ✓ | ✓ |  | ✓ |
|  | Schädle, 2020 |  | ✓ |  |  |
| Microgels | Torres, 2017 | ✓ | ✓ |  |  |
|  | Sarkar, 2016 | ✓ |  |  |  |
|  | Sarkar, 2017 | ✓ | ✓ |  | ✓ |
|  | Sarkar, 2018 | ✓ |  |  |  |
|  | Andablo-Reyes, 2019 | ✓ | ✓ |  | ✓ |
|  | Bahri, 2019 | ✓ | ✓ |  |  |
|  | Jiao, 2018 | ✓ |  |  |  |
|  | Li, 2020 | ✓ | ✓ |  |  |
|  | Araiza-Calahorra, 2019 | ✓ | ✓ |  |  |
